# Advances in Allergen Immunotherapy and Safety

**DOI:** 10.3390/vaccines13030221

**Published:** 2025-02-23

**Authors:** Samia T. Al-Shouli

**Affiliations:** Immunology Unit, Department of Pathology, College of Medicine, King Saud University, Riyadh 11461, Saudi Arabia; salshouli@ksu.edu.sa

**Keywords:** allergen immunotherapy, safety, allergen, allergy, FDA

## Abstract

Allergen immunotherapy (AIT) modifies immune responses to treat allergies. AIT treatment is a 3-month to 3-year long-term strategy, and its potential candidates are allergic rhinitis and asthma, food allergy, and insect venom allergy. AIT can be administered through specific routes recognized for allergy treatment strategies. A considerable body of knowledge about AIT is available, and the Food and Drug Administration (FDA) has approved the first peanut oral immunotherapy (OIT). The AIT effective type for other allergens and the route of administration are a real challenge. This paper reviews published literature on AIT mechanisms, administration routes, and safety.

## 1. AIT Mechanism

Leonard Noon reported AIT in 1911 and showed that pollen extract injection improved grass pollen allergy symptoms [1]. At that time, neither immunoglobulin (Ig)-E antibodies nor allergens had been characterized, and it was assumed that pollen contained a toxic substance that was responsible for the inflammatory reaction. Noon considered active immunization to induce a toxic response based on the earlier work of Dunbar in 1903 [2]. Dunbar showed pollen toxin antisera from animals neutralized “pollen toxin”. These initial findings without any details about the mode of action of pollen toxin antisera laid the AIT foundation. Unlike anti-histamines and beta-agonist suppressive drugs, AIT suppresses T-helper (TH) cells and modifies allergy patients’ immune systems to provide long-term treatment [3]. AIT induces long-term tolerance and symptom relief against allergen exposure even after treatment discontinuation [4]. In AIT, specific allergens are repeatedly injected into patients at increasing doses that induce immune response to protect against inflammatory reactions and allergy symptoms upon exposure to these particular allergens [5]. Immunized individuals’ immune systems do not respond to future allergen exposure.

**Figure 1:**
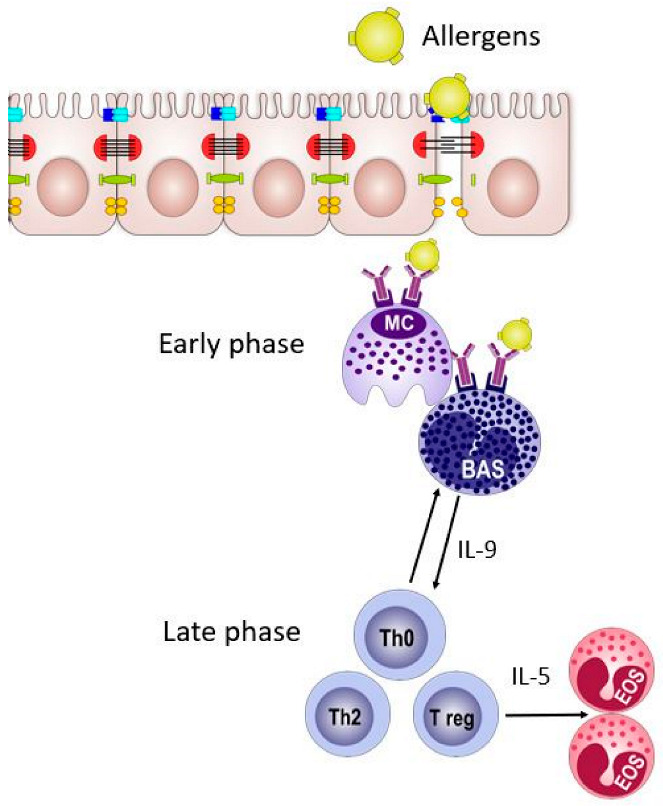
Allergen exposure to mucosal epithelia and allergy onset. Allergens enter the epithelial barrier and activate basophils. In the late phase, T cells activate eosinophils and allergy onset occurs.

Basophils and mast cells allergen exposure in the epithelial mucosa promote TH2 polarization [6] and activate group 2 innate lymphoid cells (ILC2s) [7]. Bet v 1-specific T-cells in the peripheral blood mononuclear cells (PBMC) against Bet v 1 demonstrated immunodominant T-cell epitope. Cluster of differentiation (CD)4 T-cells at the cellular level secrete interferon (IFN) and IL-10 specifically against Bet v1 [6]. The epithelial layer becomes permeable upon allergen exposure. Allergen exposure enhances epithelial layer vascular permeability and immune cell influx amplifies immune system induction [8]. In the late phase of the allergen exposure response, B cells are induced [9], which regulate nasal epithelium tight junctions (Figure 1) [7]. Later, endothelial adhesion molecules are upregulated [10], which differentiate mast cells and basophils [11]. In the final phase of allergy onset, eosinophil recruitment [12] damages the epithelium layer [13]. Allergen exposure is variable in different human tissues, and allergy development mechanisms also vary. Therefore, it is critical to consider the AIT route of administration in allergy treatment.

**Figure 2:**
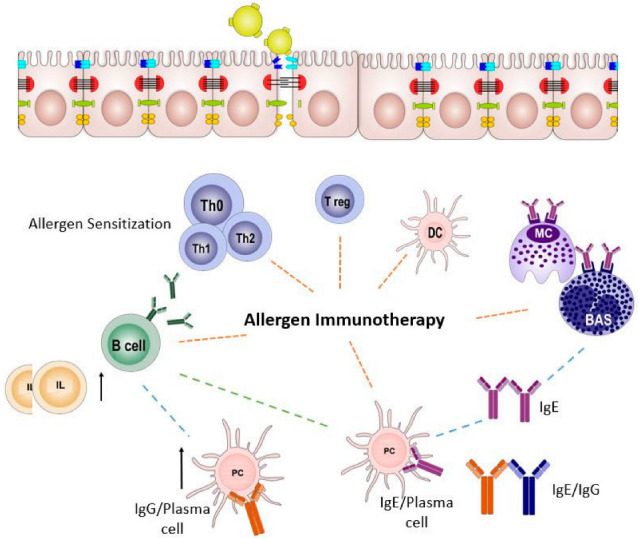
Allergy onset and allergen Immunotherapy. Allergen immunotherapy activates T cells to release interleukins and produce IgE/IgG that protects patients from future allergy episodes.

AIT suppresses allergen-specific T-helper 2 (TH2) cells, regulates B and T cells, and produces IgG- and IgA-blocking antibodies that provoke immune responses against allergen exposure (Figure 2) [14]. AIT inhibits allergen-induced thymus and chemokine production, which decreases TH2 cell accumulation in the airways. AIT enhances other regulatory T cells [15]. In a mouse model, TH2-derived interleukine-4 (IL4) causes mucosal barrier disruption, which is an important step in immune system activation [10]. Epithelial barrier disruption activates eosinophil cationic protein (ECP) and TH2 cytokines (IL4, IL5, and IL13) that amplify for 8 h and induce the immune system. AIT suppression of TH2-derived B- and T-cell regulation stops immune system activation. AIT repeated doses with increasing concentrations suppress TH2-derived cytokines and modifies and decreases basophil sensitivity for future exposure. Since Noon’s discovery, AIT has evolved successfully from subcutaneous immunotherapy (SCIT) to many other forms.

## 2. Allergy Vaccines

**Figure 3:**
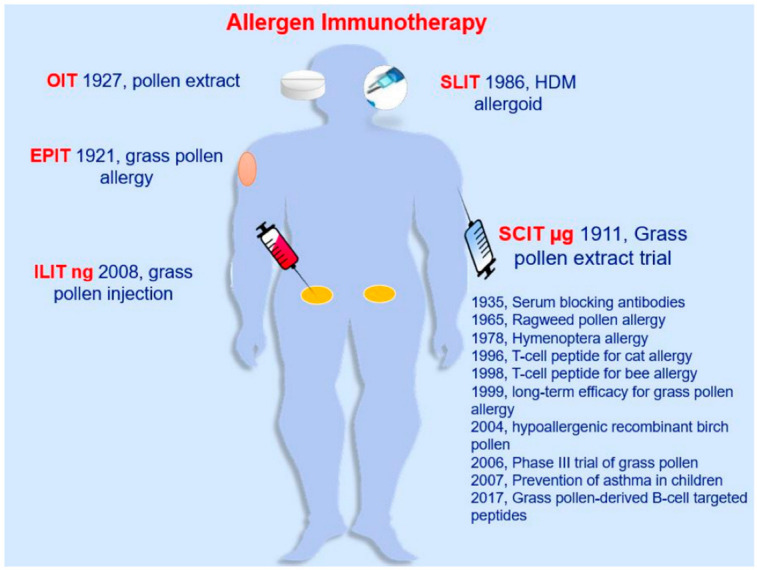
AIT history and approaches. The red acronyms show the type of AIT approach started in the mentioned year and the square brackets denote the reference.

AIT vaccines are allergen extracts from natural sources of allergens. Allergy vaccines made by different companies vary in their immunotherapy approach and mode of action. Allergen modification to create allergoids employs several strategies like structure alteration (disulfide bridge removal), recombinant proteins, large recombinant fragments production, synthetic peptides (13–71 amino acids), hydrolyzed allergens (1–10 kDa), and major allergens trimers [2,16,17]. AIT vaccines produced by different approaches differ in mode of action and efficacy. Vaccine type and batch-to-batch differences rely on the raw material purity and quality of methods employed in vaccine preparation [2]. Over time, new allergy vaccines have been developed that are administered in different doses and routes, producing variable immune responses against specific allergen exposure. For instance, AIT safely and effectively treats grass pollen allergy by modifying allergic responses and inducing long-term tolerance. Grass pollen first-year SCIT treatment in the target tissue decreased 447X basophil sensitivity [18]. The clinical efficacy of basophil sensitivity reduction after 3 weeks of treatment suggests a basophil potential predictive biomarker. Similarly, birch pollen airway inflammation in a murine model of two months of SCIT showed decreased proportions of IL5 [19]. IL5, a TH2 cytokine, is activated in response to allergen exposure. IL5 is a potential biomarker for pollen SCIT. Another form of AIT is sublingual immunotherapy (SLIT), where an immunotherapeutic dose is directly administered underneath the tongue. SLIT is reported to treat allergies effectively. Four-year SLIT prevents new allergen sensitization in respiratory allergies (12–21%), while pharmacotherapies, even if continued for a long time, do not prevent new allergen sensitization [20]. The 4-year duration is optimal for SLIT in comparison to 3-year and 5-year durations and induces 8-year immunity against respiratory allergies. The SLIT second dose induces a greater immune response than the first dose. The choice of the AIT administration route determines the immune response (Figure 3).

## 3. AIT Administration Routes

### 3.1. SCIT

Continuous subcutaneous application of allergen extract in increasing quantity for 3 years develops immunity against the target allergens. SCIT has been extensively studied and developed over time [14,21,22,23,24,25,26]. Grass pollen extract develops long-term benefits, and results are reproducible [14]. SCIT desensitizes basophils through the histamine 2 receptor (HR2), which inhibits basophil mediator release (leukotrienes, cytokines, histamine, and sulfido-peptide). SCIT inhibits IgE inhibitory mediators through IgG4 and reduces asthma clinical symptoms. Depigmented polymerized allergen vaccine in asthma SCIT and medication symptom scored 0.59 and 0.53, respectively, at a 95% confidence interval (CI). The addition of HDM to SCIT reduced β2-agonists inhalation in bronchial asthma. Modified HDM extract (depigmentation and glutaraldehyde adsorption in aluminum hydroxide polymerization) use in SCIT improved asthma symptoms [27]. SCIT treatment discontinuation suppresses sensitization to other allergens in allergic rhinitis. Thus, SCIT has clinical implications that differ from those of inhaled corticosteroids. Another modified SCIT approach incorporating hydrolyzed *Lolium perenne* pollen-allergen-extract-specific IgG75 reduced the allergen-induced conjunctival inflammation. Thymic stromal lymphopoietin (TSLP) plays a role in allergic response initiation and persistence. A human monoclonal anti-TSLP antibody (tezepelumab) improved SCIT efficacy and enhanced tolerance in allergic rhinitis patients in a 1-year therapy course [28]. SCIT shows a good response against allergen exposure but sometimes develops systemic reactions.

### 3.2. SLIT

SLIT was developed to overcome the risk of systemic reactions developed in SCIT. SLIT improves health conditions in asthma and reduces reliance on bronchodilators [29]. Randomized SLIT or inhaled corticosteroid (ICS) treatment of patients for 5 years showed that SLIT treatment had better improvement than ICS, and it suppressed rhinitis symptoms. House dust mite (HDM)-sensitized adults suffering from allergic rhinitis can be considered for HDM-SLIT if their % forced expiratory volume 1 (FEV1) is more than 70%. SLIT effectively reduced immune-mediated inflammatory responses in mite-induced rhino-conjunctivitis patients [30]. In 2023 and 2024, peanut-allergic patients SLIT (4 mg peanut protein for 4 years) showed desensitization to 800 mg effectively. The SLIT had no adverse reactions and showed efficacy of 70.2% in a double-blind placebo-controlled food challenge (DBPCFC) [31]. SLIT induces allergen-specific IgG and IgE responses in allergic patients. SLIT has been tested in phase III clinical trials and requires real-life compliance, which is much lower. SLIT tablets reduced allergic symptoms of HDM in asthmatic exacerbations [32]. However, SLIT lacks effective treatments for venom, food, and respiratory allergens (molds and cat dander).

### 3.3. Oral Immunotherapy (OIT)

OIT involves systematic allergen administration from low to high doses in the oral cavity to induce the immune system against allergens in food sources. OIT effectiveness depends on sustained unresponsiveness (SU) and desensitization. SU means the absence of clinical reaction after allergen application for a known period, while desensitization is an increase in the allergen reaction threshold to normalize against specific food allergen ingestion. However, allergen ingestion and subsequent digestion of the orally administered allergen for immunotherapy by gastric acids reduce the efficacy of this approach [14]. Digestion-resistant food allergens can be good candidates for OIT. OIT is practiced for peanut and cow’s milk in Europe and the USA [33]. OIT is practiced for cow’s milk and hen’s eggs in Japan. The FDA has approved the first OIT for peanuts [31]. Peanut OIT is administered daily in a 300 mg encapsulated whole peanut (AR101) oral product. Peanut OIT enhances the secretion of peripheral blood mononuclear cells (IL-10) and CD25+FOXP3+ T cells. OIT has shown promising results in grass pollen rhinitis for airway allergens. OIT tablet (15–25 µg *Phleum pratense* major allergen PHL p 5) perennial use for 3 years improved 30–40% grass pollen rhinitis symptoms medication scores [34,35]. Persistent long-term modification of immunologic memory in either B or T cells reduces allergy scores. Gauging the Response in Allergic rhinitis to Sublingual and Subcutaneous Immunotherapy (GRASS) study found peripheral-allergen-specific T-cell TH2 immunity suppression after SLIT and SCIT challenge [36]. The suppression reversed after three years to baseline year 3.74, but IgE-blocking activity remained active at 3 years. Allergen-specific T-cell response can induce tolerance, and B cells may have an important role in long-term protective tolerance.

### 3.4. Intra-Lymphatic Immunotherapy (ILIT)

ILIT was developed on the idea that lymph nodes have a higher concentration of immune cells. Allergen direct exposure to lymph nodes will yield better immunomodulation and IgG response than SCIT. Ultrasound-guided lymph nodes are the best option for purified allergenic peptides in nanogram application with no severe reaction for allergy treatment. The major histocompatibility complex (MHC) class I-binding peptide vaccine increased immunogenicity 10^6^ times more than intradermal and subcutaneous vaccination [37]. T-cell receptor transgenic mouse model (TCR318) intra-lymphatic injections showed more efficient results than SCIT [38]. These findings suggest that ILIT may not polarize allergen-specific responses but induce better T-regulatory responses. Similarly, bee venom allergen phospholipase A2 or Fel d 1 (cat fur allergen) direct lymph node administration increased these allergen-specific T cell and IgG responses more than subcutaneous injections. Intra-lymphatic vaccination stimulates Th1-dependent IgG2a and protects allergen-activated anaphylaxis due to enhanced antigen delivery compared to subcutaneous injection. Such immunization activates 10 times more IgG2a response with a 100 times lower allergen dose [39]. Clinical findings indicate that ILIT is safe and efficient and yields lower systemic risks. ILIT has an advantage over other AIT approaches due to low allergen doses and short duration. The three-month treatment employs three ultrasound-guided injections in lymph nodes. ILIT initial clinical trials have yielded encouraging results to optimize formulations in the future [40]. Furthermore, ILIT 3 injections during the blooming season provide relief equal to 3-year SCIT treatment [41]. ILIT is safe but requires ultrasound guidance to deliver the vaccine effectively in the lymph node, which is a cumbersome and costly process [2]. Despite ILIT’s promising results, no authorized treatment is available for routine allergy treatment and [42].

### 3.5. Epi-Cutaneous Immunotherapy (EPIT)

EPIT was reported for the first time in 1928 as a promising strategy to treat allergies [43]. EPIT also termed intradermal AIT was used for pollen allergy treatment [44]. EPIT administration on injured skin reduces allergic symptoms. Intradermal injections used in 1923 reported that three injection doses relieve patients. Specific allergens applied in patches to known grass-pollen-positive patients induced eczema and specific T-cell responses. Epi-cutaneous allergen administration may desensitize allergic asthma and reduce adverse side effects from SCIT [4]. Research on epi-cutaneous allergen application induced mild IgG rise and allergen-specific T-cell responses. Systemic peanut-allergen-specific IgG analysis confirmed that EPIT induces desensitization in peanut allergy. EPIT-induced mild IgG response to peanut allergens [2]. EPIT is based on the notion that administering an allergen through a non-vascularized epidermis will have fewer side effects. The approach uses high-dose allergens to induce the immune system against seasonal allergies. EPIT is a needle-free immunotherapy approach that is the choice of children and other patients who fear needles. EPIT can provide higher safety and efficacy and can be an alternative to SLIT and SCIT for food and aerosol allergies [45].

### 3.6. Molecular AIT for Allergy Treatment

Molecular AIT initiated with the first recombinant allergen expression, which triumphed the idea that recombinant allergens can be extract-based testing alternatives [46,47]. Molecular AIT uses recombinant vaccines for allergy treatment. The BM32 vaccine consists of four major grass pollen allergen IgE-binding site nonallergenic peptides and the hepatitis B preS protein recombination. BM32 injections induce allergen-specific IgG and improve seasonal grass pollen allergy clinical symptoms in allergic patients [48]. Recombinant allergens became available to microarray allergen chip development for multiplex IgE binding studies with human serum [49], providing opportunities for molecular AIT. Molecular AIT attempted to treat patients with allergen-derived T-cell epitopes comprising peptides and hypoallergenic derivatives. Investigators were keenly interested in improving AIT specificity and safety.

Less allergenic and safe recombinant allergen derivatives are carefully designed and used in AIT [2]. Recombinant hypoallergenic allergen derivatives containing T-cell epitopes in the AIT trial showed that genetically engineered allergen derivatives treat allergy by ameliorating allergic reactions and reducing IgE production [50]. Recombinant hypo-allergens used for specific immunotherapy can be chemically modified or genetically engineered to enhance allergen specificity, efficacy, and safety [51]. Genetic engineering approaches applied included oligomerization, fragmentation, sequence reassembly with reduced IgE reactivity, and mutation to alter allergen structure. Bet v 1 immunogenic 3 contiguous overlapping peptides 50 µg dose administration in aluminum hydroxide SCIT application showed promising results. The dose demonstrated high efficacy, fewer systemic reactions, and immunomodulatory changes in phase II b birch pollen allergic subjects [52]. All these hypo-allergens have reduced IgE reactivity and do not induce immune response except IgG-specific antibodies immunization. Molecular AIT studies prove recombinant AIT vaccines have better efficacy than extract-based AIT. These vaccines promise to induce T-cell tolerance and prevent pollen and food allergies and venoms [53].

Hypoallergenic can potentially block IgG antibodies and are vital in developing AIT vaccines. In a mouse model study, Ara h 2 mutant produced in insect-induced T-cell proliferation without inducing anaphylaxis in peanut-sensitized mice. Removal of linear and conformational epitopes from Ara h 2 reduced IgE binding and anaphylactogenic potential of the allergen but retained its T-cell activation potential [54]. Recombinant B cell allergy vaccines are good candidates for treating allergies. The BM32 grass pollen allergy recombinant B cell epitope-based vaccine was evaluated in three clinical immunotherapy studies in patients suffering from asthma and pollen allergies. Similarly, B-cell epitope-based allergy vaccines designed for cat, HDM, birch, and ragweed pollen allergy are under the initial clinical evaluation phase. These recombinant vaccines are considered safe and less allergenic [55].

## 4. Choice of Procedure

SCIT was the first reported AIT for allergies and evolved into many other forms. AIT suppresses and modifies the immune system to relieve allergenic patients against specific allergens. It is required in large concentrations (15–50 µg) and for an extended period to develop immunity against the invading allergen [34,35]. Prolonged SCIT application for 3 years effectively relieved allergy patients for an extended period after immunotherapy discontinuation [56,57]. SCIT showed 30% or more efficacy in bee venom, pollen, and HDM allergy treatment. Later on, EPIT was reported in 1928 as an alternative to SCIT injections and extended treatment [2]. EPIT administers high-dose allergens through a non-vascularized epidermis in a needle-free method. An EPIT single-patch allergen dose can be 2–21 μg, and a cumulative patch allergen dose can be 18–525 μg, which is very high [45]. EPIT has been successfully applied for grass pollen, peanut, and cow milk allergies, as well as rhinoconjunctivitis. It is advantageous over SCIT due to its injection-free method of administration and relieves children and patients who fear injections.

However, to improve AIT further and reduce invasive procedures, SLIT was reported in 1986. SLIT uses 4 mg of allergen for 36–48 months (144–192 mg cumulatively) with no adverse reactions and 70.2% efficacy. The method is a good choice for children and others who fear injection needling [31]. Another type of AIT is OIT, which administers allergens orally. Recently, the Food and Drug Administration (FDA) has approved the first OIT product (AR101, a 300 mg encapsulated Ara h 1 allergen) for peanut allergy [31]. However, orally administered allergens are at risk of gastric juice digestion, which reduces their efficacy [14]. OIT can employ only digestion-resistant food allergens. Similarly, the OIT tablet for *P. pratense* major allergen (15–25 µg PHL p 5) has 30–40% efficacy against grass pollen rhinitis [34,35]. However, all these methods provide AIT for an extended period and use high-dose allergens. There is a need to reduce the AIT period and dose. ILIT employs allergen dose in nanograms directly in the lymph node through an ultrasound-guided technique. Direct small-quantity allergen application induces 10^6^-fold stronger response than intradermal and SCIT allergen treatment. ILIT provides prolonged immunity against allergens with a shorter treatment duration [37,39]. The method showed greater efficacy but is limited by ultrasound-guided injections, making it complex and expensive [41].

All these approaches use raw/specific allergen extracts from sources, i.e., pollen, spores, and food. To improve the specificity and efficacy of AIT, IgE binding sites of non-allergenic peptides are recombined with hepatitis B preS protein. BM32 is a molecular vaccine and induces an allergen-specific IgG response against seasonal grass pollen allergy [48]. Similarly, the purified Ara h 2 mutant in *Trichoplusiani,* BTI-TN5B1-4, induces T-cell proliferation without inducing anaphylaxis in peanut-sensitized mice. Linear and conformational epitope removal from Ara h 2 reduces the allergen IgE binding and anaphylactogenic potential but retains its T-cell activation potential [54]. Several other molecular AIT vaccines are under research trials that aim to improve shortcomings in the existing AIT approaches.

## 5. AIT Safety

SLIT is safe and recommended in clinics for patients [36]. HDM allergoid SCIT application in randomized controlled trials on a larger study population showed no adverse events [58]. A one-year anti-TSLP (tezepelumab) therapy course initiated an allergic response with improved SCIT efficacy and tolerance in allergic rhinitis patients [28]. Similarly, high-dose HDM allergoids were clinically tolerable in allergic patients. SCIT tolerability was comparable to HDM SLIT tablets. The European Academy of Allergy and Clinical Immunology (EAACI) recommends HDM-SCIT for adults and children suffering from allergic asthma [59]. Improved diagnostics will help in specifying therapeutic antigens and formulations to optimize treatment response in patients. Intradermal pollen injections provide allergy symptoms relief to pollen-allergic subjects and report no adverse reactions. Initial findings mark intradermal pollen AIT as safe and efficacious [60]. ILIT induces long-lasting allergy immunity in three doses at lower concentrations in 3 months [2]. ILIT requires ultrasound-guided injections to inject therapeutic agents precisely into lymph nodes. Furthermore, ILIT has lower risks of inducing systemic risks in allergic patients. ILIT is safe and efficacious but still contains fear of injections in children and patients worrying about injections. An injection-free approach like EPIT is under consideration for patient compliance and safety.

EPIT is a new allergen administration approach and possesses higher safety. Current studies show that EPIT can provide higher safety and can be an alternative to SCIT and SLIT for pollen and food allergies [45]. EPIT showed greater safety and adherence than SLIT and OIT in children allergic to peanuts and cow milk. It is safe in children, and its efficacy is evident in younger people. Further research on EPIT may produce AIT products for clinical use. Currently, several safe AIT products are available in the market. The FDA has approved the first OIT tablets (AR101, a 300 mg encapsulated Ara h 1 allergen) for peanut allergy [31]. Similarly, four safe adjuvants are approved and are available for AIT treatments [61]. These are commonly used products microcrystalline tyrosine (MCT), aluminum hydroxide (Al (OH)3, or Alum), monophosphoryl lipid A (MPLA), and calcium phosphate (CaP).

Novel AIT-developing approaches should focus on (1) enhancing safety and efficacy, (2) AIT schedule improvement, (3) obtaining quick relief, and (4) long-term AIT cessation effects. Several groups around the globe are working on several aspects of AIT. However, major attempts fail to address these suggestions. Any future AIT attempt should consider allergen modifications and adjuvants used to suppress TH2 cells [62]. AIT products must be thoroughly reviewed for their clinical efficacy, quality, and safety. Additionally, AIT products must be well documented to describe their clinical efficacy for all relevant allergens. AIT approaches may be considered for primary prevention of IgE sensitization. So far, evidence fails to present such a feasible approach. There is a high need to explore this aspect of AIT and prevent allergic sensitization. Table 1 lists AIT safety details.

## 6. Conclusions

AIT developed in 1911 has evolved to provide greater relief precisely with less dose and greater human safety. Currently, SCIT, SLIT, EPIT, OIT, and ILIT safely provide treatment to allergic and asthmatic patients for longer-duration relief. ILIT induces long-lasting immunity in smaller allergen doses in three months but requires ultrasound-guided injections, limiting its use in clinical settings. The FDA has approved the first OIT product against peanut allergy to treat peanut-allergic patients. Other molecular-based therapies are being explored to improve AIT precision and reduce treatment time. Further research on reducing treatment time and human safety will improve treatment options for the global increasing allergy burden.

## Figures and Tables

**Table 1:** Research showing AIT safety in trials.

S. No.	Study Population	Study Duration	Status	Allergen Type	Route of Administration//Safety	Reference
1	Research	1 systemic reaction in 1000 injections 1 anaphylactic shock in 1 million injections	Before 2002, 3.4 fatal reactions/year to SCIT-related systemic allergic reactions reduced		SCIT	[63]
2	N = 24 children, median age = 9.6 years	133–795 mg peanut protein for 12 months OIT	33% of treatment group achieved 795 mg OIT	Food	OIT Safe for peanut anaphylaxis patients	[64]
3	N = 28 children age = 3–12 years	10–100 mL cow’s milk for 1 year	50% of children have negative OFC	Food	OIT Safe for lower doses Adverse reactions at higher doses	[65]
4	N = 20 mite-induced rhinoconjunctivitis patients	Symptoms score by diary cards for 2 years	Eosinophilic cationic protein serum concentration significantly decreased P = 0.04	Aeroallergen	SLIT Clinically effective for rhinoconjunctivitis	[30]
5	N =106 Mean age = 33.5	2 years grass pollen SLIT	Nasal allergen challenge	Phl p 5	SLIT No significant difference between placebo and SLIT group at 3 years follow up	[36]
6	N = 500	Analysis of pooled safety data for 6 randomized controlled trials	High-dose HDM allergoid tolerated in clinical practice	HDM allergoid	SCIT	[58]
7	N = 1085 Age = 10–65 years	Analysis of 10 RCTs	Enhanced local treatment adverse events	Food	EPIT May induce desensitization in peanut allergy	[66]
8	N = 134	3-ultrasound guided intralymphatic injections in 3 months	ILIT clinical results are encouraging	Bet v1 Fel d 1 HDM	ILIT	[42]

## Data Availability

Data were obtained from publicly available sources.
